# Risk and predictors of in-hospital mortality from COVID-19 in patients with diabetes and cardiovascular disease

**DOI:** 10.1186/s13098-020-00565-9

**Published:** 2020-07-06

**Authors:** Hadith Rastad, Hossein Karim, Hanieh-Sadat Ejtahed, Ramin Tajbakhsh, Mohammad Noorisepehr, Mehrdad Babaei, Mehdi Azimzadeh, Alireza Soleimani, Seyed Hasan Inanloo, Neda Shafiabadi Hassani, Fariba Rasanezhad, Ehsan Shahrestanaki, Zeinab Khodaparast, Hossein Golami, Mostafa Qorbani

**Affiliations:** 1 Social Determinants of Health Research Center, Alborz University of Medical Sciences, Karaj, Iran; 2 Cardiovascular Research Center, Alborz University of Medical Sciences, Karaj, Iran; 3 Endocrinology and Metabolism Research Center, Endocrinology and Metabolism Clinical Sciences Institute, Tehran University of Medical Sciences, Tehran, Iran; 4 Obesity and Eating Habits Research Center, Endocrinology and Metabolism Clinical Sciences Institute, Tehran University of Medical Sciences, Tehran, Iran; 5 Non-Communicable Diseases Research Center, Alborz University of Medical Sciences, Karaj, Iran; 6 Research Center for Health, Safety and Environment (RCHSE), Alborz University of Medical Sciences, Karaj, Iran; 7 Dietary Supplements and Probiotic Research Center, Alborz University of Medical Sciences, Karaj, Iran; 8 Chronic Diseases Research Center, Endocrinology and Metabolism Population Sciences Institute, Endocrinology and Metabolism Research Institute, Tehran University of Medical Sciences, Tehran, Iran

**Keywords:** COVID-19, Diabetes, Cardiovascular diseases, Death

## Abstract

**Background:**

Diabetes mellitus (DM) and cardiovascular disease (CVD) are present in a large number of patients with novel Coronavirus disease 2019 (COVID-19). We aimed to determine the risk and predictors of in-hospital mortality from COVID-19 in patients with DM and CVD.

**Methods:**

This retrospective cohort study included hospitalized patients aged ≥ 18 years with confirmed COVID-19 in Alborz province, Iran, from 20 February 2020 to 25 March 2020. Data on demographic, clinical and outcome (in-hospital mortality) data were obtained from electronic medical records. Self-reported comorbidities were classified into the following groups: “DM” (having DM with or without other comorbidities), “only DM” (having DM without other comorbidities), “CVD” (having CVD with or without other comorbidities), “only CVD” (having CVD without other comorbidities), and “having any comorbidity”. Multivariate logistic regression models were fitted to quantify the risk and predictors of in-hospital mortality from COVID-19 in patients with these comorbidities.

**Results:**

Among 2957 patients with COVID-19, 2656 were discharged as cured, and 301 died. In multivariate model, DM (OR: 1.62 (95% CI 1.14–2.30)) and only DM (1.69 (1.05–2.74)) increased the risk of death from COVID-19; but, both CVD and only CVD showed non-significant associations (p > 0.05). Moreover, “having any comorbidities” increased the risk of in-hospital mortality from COVID-19 **(**OR: 2.66 (95% CI 2.09–3.40)). Significant predictors of mortality from COVID-19 in patients with DM were lymphocyte count, creatinine and C-reactive protein (CRP) level (all P-values < 0.05).

**Conclusions:**

Our findings suggest that diabetic patients have an increased risk of in-hospital mortality following COVID-19; also, lymphocyte count, creatinine and CRP concentrations could be considered as significant predictors for the death of COVID-19 in these patients.

## Background

Coronavirus disease 2019 (COVID-19) caused by a novel coronavirus, severe acute respiratory syndrome coronavirus 2 (SARS-CoV-2), [1] has led to substantial morbidity and mortality worldwide since the first report of COVID-19 in December 2019. The more severe form of diseases leading to death is supposed to occur more frequently in older patients and those who have some underlying comorbidities [2, 3]. Among comorbidities, diabetes mellitus (DM) and Cardiovascular Disease (CVD) both are present in a large number of patients with novel Coronavirus disease 2019 (COVID-19) [3–5].

Few previous studies showed that DM and CVD as underlying comorbidities might increase the risk of death in patients with COVID-19; [6–10] but, they have failed to provide robust evidence on these associations because of too small sample size, which led to the low precision of the estimations, and lack of taking confounding factors into consideration [4, 10]. Besides, none of the previous studies mainly focused on the effect of DM and CVD on the death of COVID-19 and none assessed these associations stratified by age, gender and diagnostic criteria of COVID-19 [6–9, 11]. A recent study using data from 174 patients with COVID-19 showed that diabetic patients without other comorbidities are at a higher risk for severe pneumonia, death, as well as release of tissue injury related enzymes, and excessive inflammation responses [10].

Therefore, it is still unknown the extent to which DM or CVD, alone and in combination with other comorbidities, might put patients with COVID-19 at the increased risk of mortality. Besides, the factors that may predict the severity and death of the COVID-19 in patients with DM or CVD is unidentified [12].

Hence, the present study aimed to determine the risk and predictors of in-hospital mortality from COVID-19 in patients with DM and CVD in Alborz, Iran.

## Methods

### Study design and participants

In this retrospective cohort study, we included all adult inpatients (≥ 18 years old) with radiological-confirmed COVID-19 admitted between 20 February 2020 and 25 March 2020 in Alborz province, Iran. We excluded patients who were still hospitalized in this survey.

### Data collection

We extracted data on demographic and clinical characteristics (including age, gender, medical history, history of exposure to people with confirmed SARS-CoV-2 infection, having any comorbidities (self-reported), signs, symptoms, O2 saturation, and being ventilated) and laboratory findings of each patient at the first day of hospital admission, the date of hospital admission and discharged [dead or alive (cured)], real-time polymerase chain reaction (RT-PCR) test, and chest computed tomography (CT) imaging from the electronic medical record system of all hospitals of the Alborz province, Iran (n = 18).

This research was conducted according to the Declaration of Helsinki guidelines. Research and Ethics Committee of Alborz University of Medical Sciences (ABZUMS) approved the study and waived the requirement for informed consent. An identification number unique was assigned to each patient to protect confidentiality and anonymity.

### Definitions

#### Radiological diagnosis of COVID-19 disease

The diagnosis was according to the Iranian Society of Radiology COVID-19 Consultant Group (ISRCC) criteria [13]. The diagnosis was based on having clinical symptoms of COVID-19 infection, including fever (axillary temperature of at least 37.3 °C) or respiratory symptoms (cough or shortness of breath), with a positive pulmonary abnormality on chest CT according to the radiological criteria of COVID-9 infection. Since chest CT imaging is a more reliable, feasible, and rapid method to diagnose and assess COVID-19 in comparison to RT-PCR, especially in epidemic regions like Iran [13, 14], it is routinely utilized as a primary and more sensitive tool for diagnosis of COVID-19 in our country. All inpatients underwent a Chest CT scan on admission.

#### Laboratory testing

The oropharyngeal swab specimens of all patients were collected and examined in predetermined laboratories across the province to detect SARS-CoV-2 viral nucleic acid using RT-PCR assay. Among all included patients, patients with positive RT–PCR test were defined as laboratory-confirmed patients.

Medical laboratory findings, including the counts of white blood cells (WBC), neutrophils and lymphocytes; serum concentrations of, creatinine, lactate dehydrogenase (LDH), albumin, aspartate and alanine transaminases (AST, ALT), hemoglobin (Hb), Erythrocyte Sedimentation Rate (ESR), and C-reactive protein (CRP) were collected for each patient.

### Outcome

The outcome of interest was in-hospital mortality following COVID-19 infection. The study population was classified into two groups: discharged as cured (survivors) or dead (non-survivors). Patients were discharged from hospital based on the following criteria: lack of fever for at least 72 h, clinical alleviation of respiratory symptoms, and improvement in pulmonary abnormalities on chest CT imaging.

### Comorbidity

On admission, patients were asked if they had a history of physician diagnosis (of) or medication use for the comorbidities listed below: DM, CVD, cancer, chronic renal failure (CRF) (dialysis or non-dialysis), chronic liver diseases, psychological disorder, chronic respiratory disease, asthma, thyroid dysfunction, immunodeficiency, autoimmune disease, hematologic disease, and neurological disorder. In the first part we divided all patients according to whether they had DM/CVD. In the second part to explore the pure effect of DM/CVD part we excluded patients with comorbidities other than DM/CVD. To address these two aims we classified comorbidities into six groups: DM (having DM with or without other comorbidities), “only DM” (having DM without other comorbidities), CVD (having CVD with or without other comorbidities), “only CVD” (having CVD without other comorbidities), “CVD or DM”, and the “presence of any comorbidity”.

### Statistical analysis

The normality of continuous variables was assessed using the Kolmogorov–Smirnov test. Continuous variables with and without normal distribution were reported as mean (standard deviation (SD)) and median (interquartile range (IQR)), respectively. Categorical variables were presented as number (percentage). Continuous variables with or without normal distribution between survivor and non-survivor were compared using t-test and Mann–Whitney U test, respectively. Comparisons of categorical variables between survivors and non-survivors patients were performed using Chi squares tests.

Univariate and multivariate logistic regression analyses were performed to explore the association of underlying comorbidities with the risk of in-hospital mortality. In the multivariate model, gender, age and laboratory tests were adjusted as potential confounders. The results of logistic regression analysis were presented as odds ratios (OR) with corresponding 95% confidence intervals (CIs). Stratified analysis was performed according to age (< 65 and ≥ 65 years) and gender groups. Forest plot was used to illustrate the results of multivariate logistic regression analysis according to age and gender groups schematically. To evaluate the effect of excluding negative RT-PCR on our main findings, we conducted a sensitivity analysis focusing on only laboratory-confirmed patients (positive RT-PCR). A P-value of less than 0.05 was considered as statistically significant. Statistical analyses were done using SPSS Version19.0 (SPSS Chicago, IL, USA) or STATA version11 (Stata Corp LP, College Station, TX, USA).

## Results

A total of 2957 radiological-confirmed COVID-19 patients were included in the present study, of whom, 1412 patients were confirmed by RT-PCR (laboratory-confirmed rate: 47.7%). In terms of the outcome, 2656 (89.8%) were discharged as cured and 301 (10.2%) died during hospitalization. The mean age (SD) was 54.8 (16.9), and 1589 (53.7%) patients were male. The most common symptoms on admission were cough (56.4%), followed by shortness of breath (49.2%) and fever (43.5%). Overall, one or more comorbidities were present in 44.5% (134) of patients; CVD and DM were present in 10.6% (314) and 9.0% (267) of patients, respectively. In survivors, the median time (IQR) of general ward and ICU stay were 5 days (3–7) and 9 days (7–11), respectively. In non-survivors, the median time (IQR) from admission to death was 4 days (2–6). Table 1 shows general characteristics and disease-related symptoms in included patients according to survival status.

**Table 1:** General characteristics and disease-related symptoms in the study population

Characteristics	Total, N = 2957	Non-survivors, N = 301	Survivors, N = 2656	P-value
Age Mean (sd)	54.8 (16.9)	67.3 (15.8)	53.3 (16.4)	< 0.001
Gender, % (N)				
Male	53.7% (1589)	55.5% (167)	53.5% (1422)	0.522
Female	46.3% (1368)	44.5% (134)	46.5% (1234)	
Symptoms, % (N)				
Caught	56.4% (1667)	42.9% (129)	57.9% (1538)	< 0.001
Fever	43.5% (1287)	42.9% (129)	43.6% (1158)	0.809
Shortness of breath	49.2% (1455)	60.1% (181)	48.0% (1274)	< 0.001
Tiredness	16.9% (499)	7.3% (22)	18.0 (477)	< 0.001
Ventilated (Yes), % (N)	7.0% (208)	48.2% (145)	2.4% (63)	< 0.001
O_2_ saturation < 93%, % (N)	43.6% (1288)	78.7% (237)	39.6% (1051)	< 0.001

**Fig. 1:**
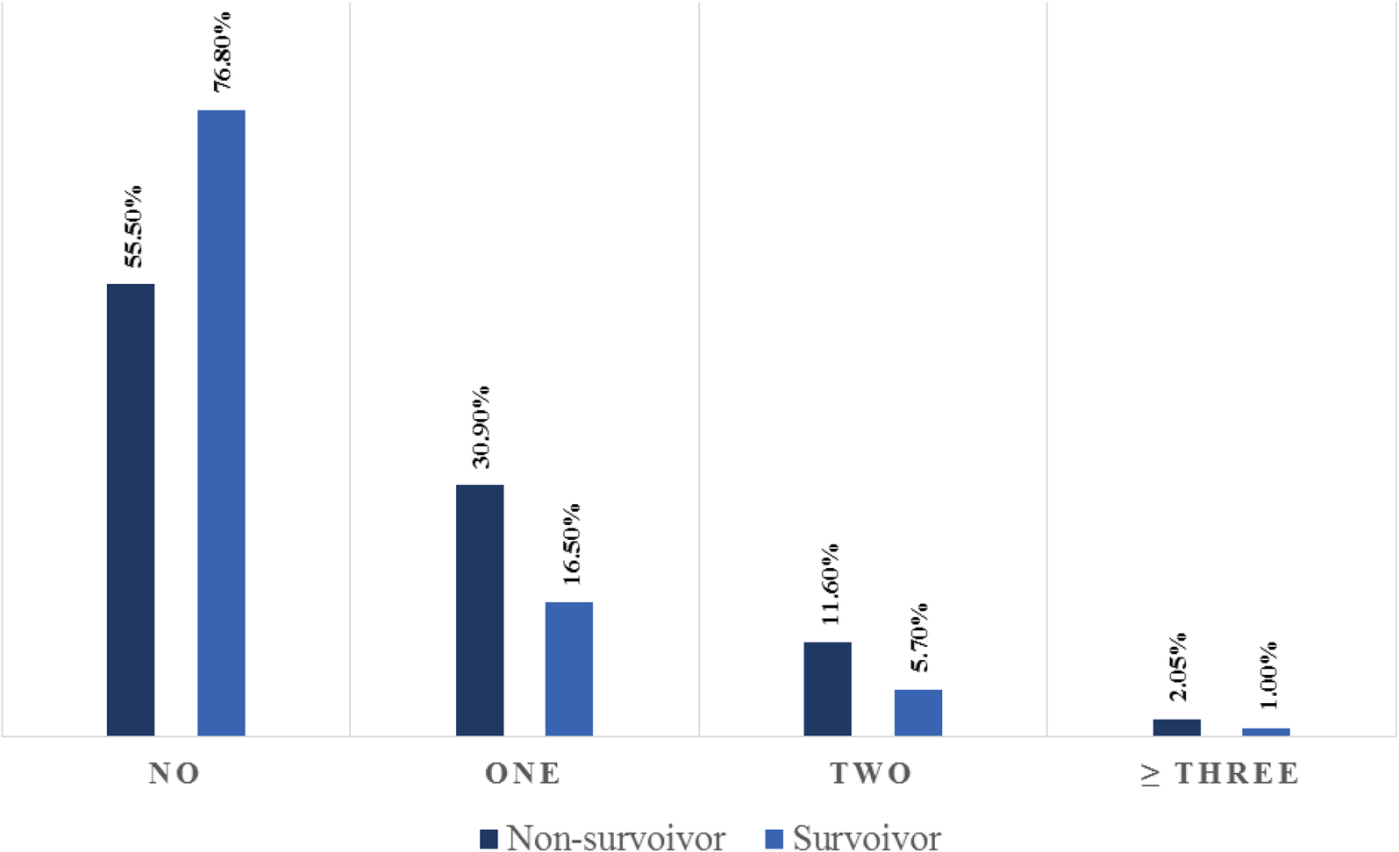
The number of comorbidities in patient with COVID-19 by survival status

**Fig. 2:**
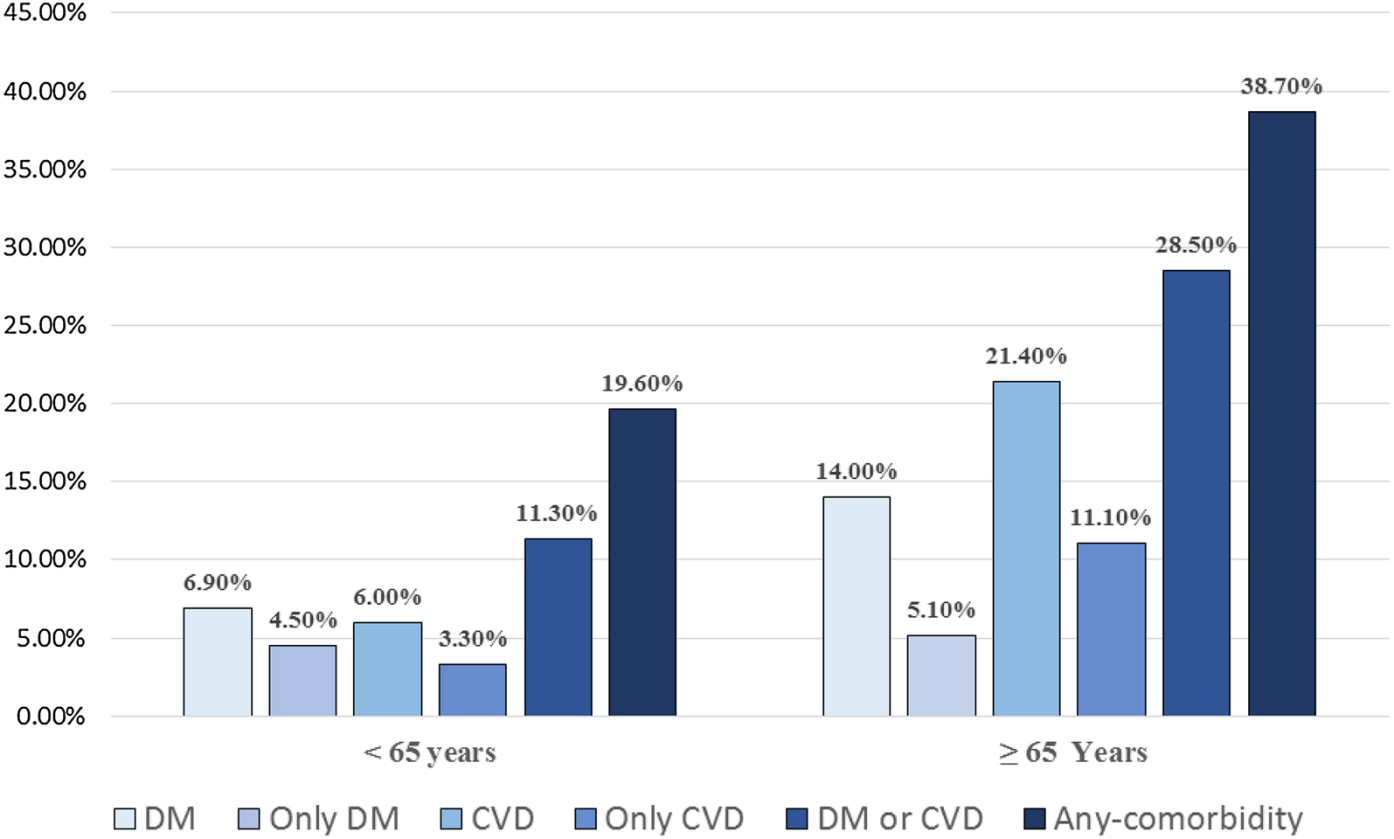
The prevalence of comorbidities in patient with COVID-19 by age group. DM (all): DM with or without other comorbidities. “Only DM”: DM without other comorbidities. CVD (all): CVD with or without other comorbidities. “Only CVD”: CVD without other comorbidities. “Any comorbidity”: The presence of any comorbidity

**Fig. 3:**
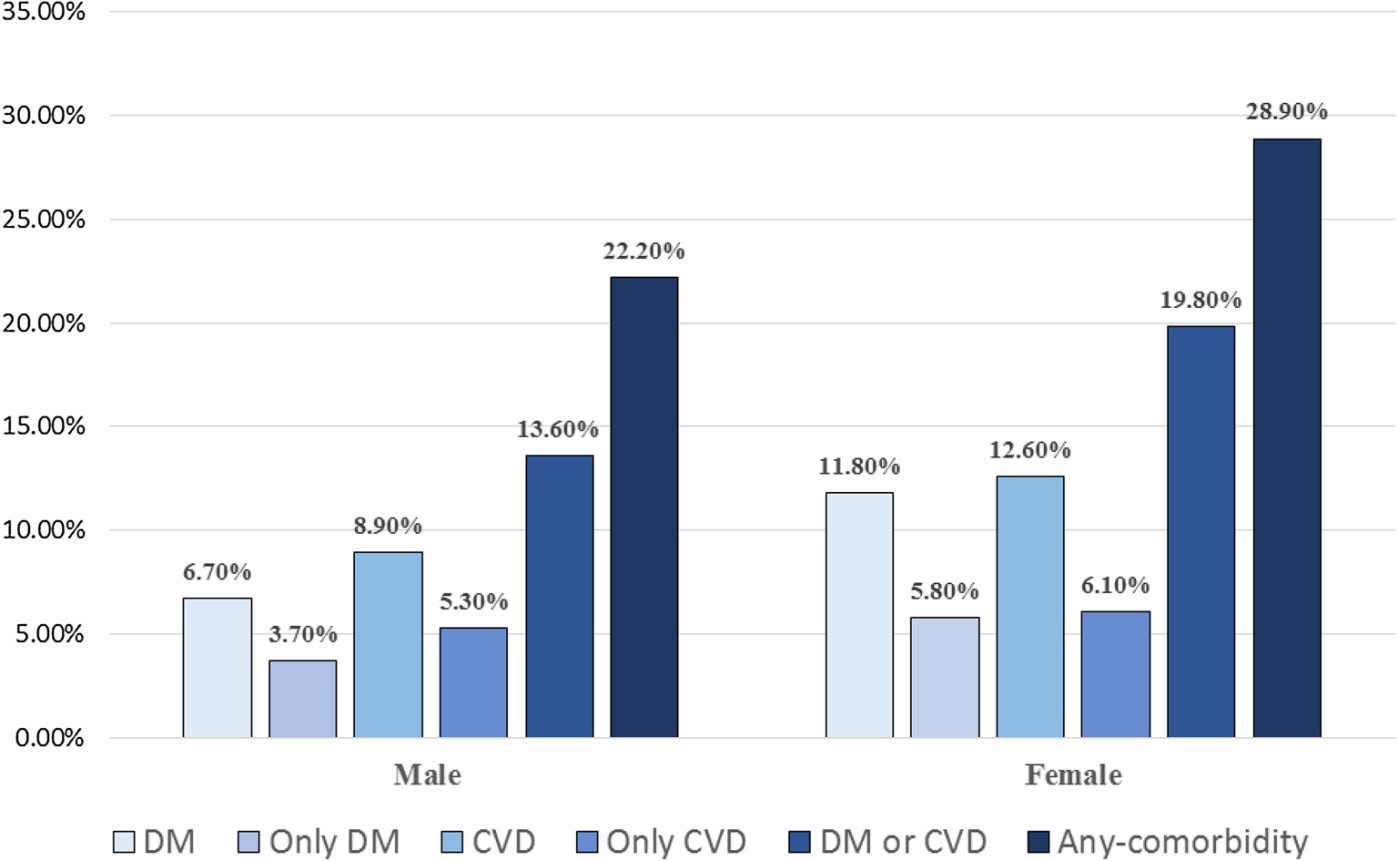
The prevalence of comorbidities in patient with COVID-19 by Sex group. DM (all): DM with or without other comorbidities. “Only DM”: DM without other comorbidities. CVD (all): CVD with or without other comorbidities. “Only CVD”: CVD without other comorbidities. “Any comorbidity”: The presence of any comorbidity

Overall, non-survivors significantly were older, more likely to present with O2 saturation < 93%, and receive invasive mechanical ventilation on admission than survivors (all P-values < 0.05). Besides, non-survivors more frequently presented with a complaint of shortness of breath, but less frequently aught and tiredness compared to survivors. A higher percentage of non-survivors had at least one comorbidity compared to survivors (Table 1; Fig. 1). The prevalence of the assessed comorbidities was higher in patients older than 65 years and females than other patients (Figs. 2, 3).

Table 2 presents laboratory findings on admission in all patients, overall and by survival status. Compared to survivors, non-survivors significantly had a lower lymphocyte count (median (IQR): 1.1 (0.6–1.7) vs. 2.5 (1.7–3.5), P-value < 0.001), but a higher count of WBC [8.3 (5.4–12.0) vs. 5.5 (4.3–7.2), P-value < 0.001] and neutrophil [8.0 (7.1–8.7) vs. 6.9 (6.0–7.7)]. Also, a higher concentration of AST, serum creatinine, CRP, and LDH, but a lower concentration of Albumin was observed in non-survivors than survivors (all P-values < 0.05).

**Table 2:** Laboratory findings on admission and presence of the comorbidities in study population, overall and by survivor status

Characteristics	Total median (IQR)	Non-survivors median (IQR)	Survivors median (IQR)	P-value
WBC count, × 10^9^ per L,	5.6 (4.4–7.7)	8.3 (5.4–12.0)	5.5 (4.3–7.2)	*<0.001*
Lymphocyte count, × 10^9^/L	2.3 (1.4–3.3)	1.1 (0.6–1.7)	2.5 (1.7–3.5)	*<0.001*
Neutrophils, × 10^9^/Ml	7.0 (6.1–7.9)	8.0 (7.1–8.7)	6.9 (6.0–7.7)	*<0.001*
Albumin, g/L	20.6 (13.0–27.0)	12.0 (6.0–19.0)	21.0 (15.0–28.0)	*<0.001*
AST, U/L	37.0 (29.0–49.0)	45.5–35.3–62.0)	36.0 (28.0–47.0)	*<0.001*
ALT, U/L	31.0 (23.0–42.0)	35.0–25.0–53.0)	31.0 (22.0–41.0)	0.281
Creatinine, mg/dl	1.02 (0.9–1.2)	1.20 (1.00–1.74)	1.00 (0.90–1.20)	*<0.001*
LDH, U/L	459 (366–576)	578 (423–738)	442 (363–559)	*0.006*
Hb, g/dL	13.9 (12.5–15.0)	13.3 (11.5–14.9)	13.9 (12.6–15.0)	0.146
Esr, mm/h	45.0 (27.0–65.0)	53.0 (31.3–77.3)	44.0 (27.0–65.0)	0185
CRP, mg/l	18 (2–64)	45 (3–106)	16 (2–56)	*0.008*
Comorbidities				
DM (all)	9.0% (267)	15.9 (48)	8.2% (219)	< 0.001
Only DM	4.7% (138)	7.6% (23)	4.3% (115)	0.010
CVD (all)	10.6% (314)	17.9% (54)	9.8% (260)	< 0.001
Only CVD	5.7% (168)	9.0% (27)	5.3% (141)	0.009
DM or CVD	16.5% (487)	28.9% (87)	15.1% (400)	< 0.001
Any comorbidity	25.3% (749)	44.5% (134)	23.2% (615)	< 0.001

DM (all): DM with or without other comorbidities. “Only DM”: DM without other comorbidities. CVD (all): CVD with or without other comorbidities. “Only CVD”: CVD without other comorbidities. “Any comorbidity”: The presence of any comorbidity

*IQR* inter quartile range, *ALT* alanine transaminases, *AST* aspartate transaminases, *CRP* C-reactive protein, *Esr* erythrocyte sedimentation rate, *Hb* hemoglobin, *LDH* lactate dehydrogenase, *PT* prothrombin time, *WBC* white blood cell, *CVD* cardiovascular diseases, *DM* diabetes mellitus

All assessed comorbidities including DM, only DM, CVD, only CVD, DM or CVD, and “the presence of any comorbidity” were significantly more prevalent in non-survivors than survivors (all P-values < 0.05) (Table 2). In patients with positive RT-PCR the same pattern was observed between non-survivors and survivors from COVID-19 (Additional file 1: Table S1).

Table 3 presents the results of logistic regression models. In univariable analysis, DM (OR (95% CI); 2.11 (1.51–2.96), only DM (1.83 (1.15–2.91)), CVD (2.02 (1.46–2.78)), only CVD (1.76 (1.14–2.70)), “DM or CVD” (2.29 (1.75–3.0)), and “having any comorbidities” (2.66 (2.09–3.40)) increased the odds of in-hospital death. In the multivariate model, after adjusting for gender (Model II), all assessed comorbidities continue to be a significant risk factor for in-hospital mortality (all p-values < 0.05). When age was additionally adjusted; the association of DM (1.62 (1.14–2.30)), only DM (1.69 (1.05–2.74)), “DM or CVD” (1.49 (1.12–1.98)), and “the presence of any comorbidities” (1.49 (1.12–1.98)) with in-hospital death remained statistically significant; but, this significance was lost for the association of CVD and only CVD with in-hospital death (both P-values > 0.05).

**Table 3:** Risk of mortality due to comorbidities in patients with COVID-19: logistic regression model

Characteristics	Model I^a^ OR (95% CI)	Model II^b^ OR (95% CI)	Model III^c^ OR (95% CI)
DM (all)	2.11 (1.51–2.96)^§^	2.15 (1.53–3.03)^§^	1.62 (1.14–2.30)^§^
Only DM	1.83 (1.15–2.91)^§^	1.84 (1.16–2.94)^§^	1.69 (1.05–2.74)^§^
CVD	2.02 (1.46–2.78)^§^	2.04 (1.48–2.81)^§^	1.17 (0.83–1.64)
Only CVD	1.76 (1.14–2.70)^§^	1.76 (1.15–2.71)^§^	0.99 (0.63–1.54)
DM or CVD	2.29 (1.75–3.0)^§^	2.33 (1.78–3.06)^§^	1.49 (1.12–1.98)^§^
Any comorbidity	2.66 (2.09–3.40)^§^	2.70 (2.11––3.46)^§^	1.86 (1.44–2.40)^§^

DM (all): DM with or without other comorbidities. “Only DM”: DM without other comorbidities. CVD (all): CVD with or without other comorbidities. “Only CVD”: CVD without other comorbidities. “Any comorbidity”: The presence of any comorbidity

*CVD* cardiovascular diseases, *DM* diabetes mellitus, *OR* odds ratio, *CI* confidence interval

^§^ P < 0.05

^a^Crude, ^b^ adjusted for gender, ^c^ additionally adjusted for age and laboratory tests

In the sensitivity analysis, after excluding the patients with negative PCR, we found the similar significant results on the association of DM, only DM, and any comorbidities with in-hospital death. However, the association of “DM or CVD” with in-hospital death did not reach to statistically significant level in the multivariate model (Additional file 1: Table S2).

Table 4 shows predictors of mortality of the COVID-19 in patients with DM and CVD based on the results of logistic regression models. In the adjusted models, significant predictors of mortality in patients with DM were lymphocyte count, creatinine and CRP concentrations, and in patients with CVD were age, lymphocyte count, and albumin concentrations.

**Table 4:** Predicting factors for death of the COVID-19 in patients with DM, only DM, CVD, and only CVD

Variable	In patients with DM (all), N = 267		In patients with CVD, N = 314		In patients with only CVD, N = 168		In patients with only DM, N = 138	
	Crude OR OR (95% CI)	Adjusted^†^ OR (95% CI)	Crude OR OR (95% CI)	Adjusted^†^ OR (95% CI)	Crude OR OR (95% CI)	Adjusted^†^ OR (95% CI)	Crude OR^†^ OR (95% CI)	Addjusted^†^ OR (95% CI)
Age	1.06 (1.03–1.08)*	–	1.05 (1.02–1.07)*	1.22 (1.06–1.41)*	1.04 (1.01–1.08)*	1.11 (1.02–1.20)*	1.04 (1.00–1.07)	
Sex (M/F)	1.17 (0.62–2.20)	–	1.37 (0.78–2.44)	–	1.43 (0.64–3.16)		1.88 (0.72–4.91)^#^	
Presence of Other comorbidities	1.26 (0.67–2.34)	–	0.99 (0.56–1.78)	–	–	–	–	–
Laboratory finding								
WBC count, × 10^9^ per L,	1.54 (1.23–1.92)*	–	1.35 (1.16–1.58)*	–	1.04 (0.97–1.12)	–	1.34 (1.03–1.76)*	
Lymphocyte count, × 10^9^/L	0.58 (0.51–0.66)*	0.81 (0.71–0.93)*	0.63 (0.56–0.68)*	0.71 (0.54–0.93)*	0.62 (0.54–0.71)*	0.68 (0.56–0.81)*	0.59 (*0.49–0.71)	0.74 (0.59–0.94)*
Neutrophils, × 10^9^/Ml	1.21 (1.13–1.29)*	–	1.28 (1.19–1.37)*	–	1.47 (1.29–1.67)*		1.18 (1.09–1.28)*	–
AST, U/L	1.17 (1.10–1.24)*	–	1.40 (1.29–1.51)*	1.15 (0.99–1.37)	1.48 (1.32–1.66)*		1.05 (1.00–1.10)^#^	–
ALT, U/L	1.03 (0.99–1.05)^#^	–	1.33 (1.18–1.49)		1.32 (1.10–1.60)*		1.00 (0.99–1.03)^#^	–
Albumin, g/L	0.64 (0.58–0.71)*	–	0.56 (0.50–0.64)	0.68 (0.50–0.92)*	0.61 (0.53–0.70)*	0.76 (0.53–0.86)*	0.75 (0.67–0.83)*	–
Creatinine, mg/dl	1.46 (1.23–1.78)*	12.72 (1.87–86.70)*	1.03 (0.65–1.61)	–	1.27 (0.25–6.32)		2.02 (0.72–5.7)^#^	–
LDH, U/L	102 (1.01–1.03)*	–	1.03 (1.02–1.04)*	–	1.02 (1.01–1.03)		1.02 (1.01–1.03)*	–
CRP	1.09 (1.07–1.11)*	1.02 (1.0–1.04)	1.13 (1.10–1.55)	–	1.14 (1.09–1.18)*	1.09 (1.04–1.14)*	1.08 (1.05–1.11)*	1.03 (1.00–1.06)
Esr, mm/h	1.04 (1.01–1.06)*	–	1.05 (1.02–1.08)	–	1.05 (1.01–1.10)*		1.02 (0.99–1.05)^#^	–
Hb, g/dL	0.54 (0.40–0.75)*	–	0.39 (0.27–0.57)*	–	0.28 (0.15–0.52)*		0.61 (0.42–90)*	–

DM (all): DM with or without other comorbidities. “Only DM”: DM without other comorbidities. CVD (all): CVD with or without other comorbidities. “Only CVD”: CVD without other comorbidities. “Any comorbidity”: The presence of any comorbidity

*OR* odds ratio, *CI* confidence interval, *CVD* cardiovascular diseases, *DM* diabetes mellitus, *IQR* inter quartile range, *ALT* alanine transaminases, *AST* aspartate transaminases, *CRP* C-reactive protein, *Esr* erythrocyte sedimentation rate, *Hb* Hemoglobin, *LDH* lactate dehydrogenase, *PT* prothrombin time, *WBC* white blood cell

*P-value < 0.05; ^#^ P-value < 0.20; ^†^ all variables with P < 0.2 in the univariate model were included in multivariate model

**Fig. 4:**
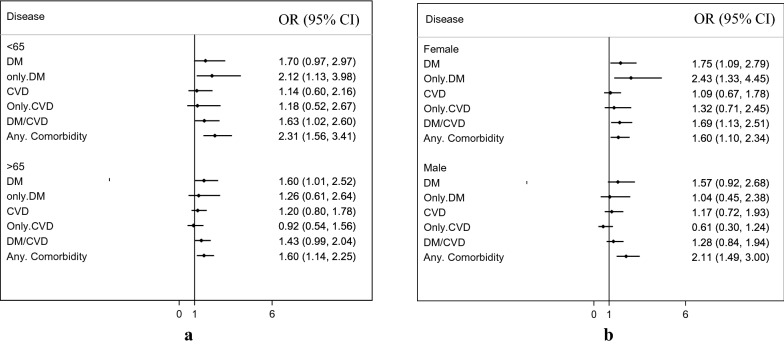
Forest plot of the multivariate association between the comorbidities and in-hospital mortality by age group (**a**; adjusted for age in year and gender) and gender (**b**; adjusted for age). DM (all): DM with or without other comorbidities. “Only DM”: DM without other comorbidities. CVD (all): CVD with or without other comorbidities. “Only CVD”: CVD without other comorbidities. “Any comorbidity”: The presence of any comorbidity

Figures 4 schematically represent the results of the multivariate logistic regression model for associations of the comorbidities with in-hospital mortality according to gender and age groups (< 65 years and ≥ 65 years). In stratified analyses, the significant associations between DM and only DM with in-hospital mortalities were observed for female patients and younger patients. Additional file 1: Figure S1 presents schematically the multivariate association between the comorbidities and in-hospital mortality of COVID-19 by age and sex group in patients with positive RT-PCR.

## Discussion

In quantifying the risk of mortality due to comorbidity in patients with COVID-19, we showed that DM increased the odds of death significantly in all patients. This finding was concordant with previous studies [4, 10, 11, 15, 16]. On the other hand, the association of CVD with COVID-19 mortality lost its significance in the multivariate model after adjusting for age. It shows the great importance of age in morbidity and mortality of cardiovascular complications, which could be justified by the strong correlation of age and CVD incidence. We also conducted a sensitivity analysis, and we showed that DM remained a significant risk factor for mortality in patients with laboratory-confirmed COVID-19. Zhou et al. study showed that odds of in-hospital death was higher in patients with DM and coronary heart disease (CHD), and older age was determined as a risk factor for death in adult patients with COVID-19 [15]. Moreover, we showed that diabetic patients without other comorbidities (only diabetes) were at a higher risk of mortality. This finding was more similar to Ghou et al., study [10]. Using data from 174 COVID-19 patients in China, they found that diabetic patients without other comorbidities (n = 24) are at a higher risk for severe pneumonia, death, as well as release of tissue injury related enzymes, and excessive inflammation responses [10].

Based on our finding, lymphocyte count, creatinine and CRP level were significant predictors for death of COVID-19 in diabetic patients. While lymphocyte count was inversely associated with the death following COVID-19, creatinine and CRP level had direct association with it.

The defect of cellular immune response and cytokine storm may play roles in the development of acute respiratory distress syndrome [17]. Since diabetic patients suffer from a less robust immune system due to chronic hyperglycemic and inflammatory states, DM could be a risk factor for COVID-19 progression and death [17–20]. Moreover, there are conflicting results regarding the use of angiotensin-converting enzyme 2 (ACE2)-increasing drugs in COVID-19 patients [20, 21]. Some studies proposed the harmful effects of these drugs on infection severity while the other ones found the drugs useful for preventing pneumonia and the risk of mortality [16, 22]. Fang et al. study suggest that patients who are treated with ACE2-increasing drugs are at higher risk for severe COVID-19 infection and, therefore, should be monitored for ACE2-modulating medications, such as ACE inhibitors [16]. Although in a retrospective cohort study it has been found that there were no association between use of ACE2-increasing drugs and COVID-19 test positivity [23], more studies are needed regarding the effect of these drugs on COVID-19 severity.

Based on our findings, the presence of DM might predispose patients with COVID-19 to develop a more severe form of the disease, leading to the worst consequences and death. It could be explained by the weak immune system in diabetic individuals, especially in those with poor glycemic control [24, 25]. This finding highlights that, in the case of access to a safe and efficient vaccine against the COVID-19 virus, these patients should be put in higher priority as a high-risk group.

In stratified analysis, we reanalyzed the data for gender and age groups, and we showed that DM only in females and patients younger than 65 years increased the risk of death. Previous observations confirmed the age and gender differences in glycemic control and treatment responses in diabetic patients [26–30]. According to the evidence, women with DM are less likely to reach the ideal level of hemoglobin A1c (HbA1c) compared with men [26, 27, 30]. The risk of all-cause mortality was higher in females with DM. Moreover, surveys showed that younger adults have poorer glycemic control compared to older diabetic patients [28, 29]. These issues are of great importance in interpreting the findings of epidemiological studies. Furthermore, Bello-Chavolla et al. declared early-onset diabetes and obesity as risk factors for mortality in COVID-19 patients in Mexico [31]. Therefore, newly diagnosed diabetes mellitus with uncontrolled hyperglycemia may be linked to increased risk of COVID-19 fatality. On the other hand, since ACE2 receptors are expressed in pancreatic beta cells, it is plausible that SARS-CoV-2 cause alterations in glucose metabolism which result in complication of the pathophysiology of preexisting diabetes or new-onset diabetes [31]. Although it has been shown that obesity mediates 49.5% of the COVID-19 lethality which attributed to diabetes in Mexican population [32], we had no data on weight status of patients for adjustment. To date, the exact mechanisms underlying strong association between obesity and COVID-19 severity were not clarified. However, it may be the consequence of low-grade chronic inflammation and suppressed immunity in obese persons [33].

In the present study, out of 2957 adult COVID-19 hospitalized patients, 1412 patients were laboratory-confirmed COVID-19. In our study, the diagnostic criteria of COVID-19 were based on abnormal chest CT scans as well as clinical manifestations of infection, and not all the cases had positive RT-PCR test may be due to low sensitivity of the test. Moreover, we did this test only one time without any repetitions, so we believed that detection according to chest CT and clinical symptoms of infection could be more reliable. It should be noted that a report of 1014 cases in China also showed that chest CT had higher sensitivity for the diagnosis of COVID-19 as compared with initial RT-PCR from swab samples [14].

In the present study, the most common symptoms on admission were cough, followed by shortness of breath and fever. The most common symptoms on admission were also reported fever and cough in Wuhan, China [15]. In comparison with survivors, non-survivor were older, and a higher percentage of them were presented with shortness of breath, O_2_ saturation < 93%, and required invasive mechanical ventilation on admission.

According to the previous investigations, older age has been nominated as an important risk factor for mortality in SARS and Middle East respiratory syndrome (MERS) [33–35]. Consistent with our observation, studies on COVID-19 have also reported that the increase in age was associated with a high mortality rate [15, 36]. The age-dependent defects in immune cell function and increased production of inflammatory cytokines may cause a poor immune response in the control of viral replication and result in poor outcomes [37].

In this study, CVD and DM were the most common comorbidities seen in 10.6% and 9.0% of infected patients. In a meta-analysis of eight studies with 46,248 infected patients, the most prevalent comorbidities among patients were hypertension (17%), DM (8%), and CVD (5%) [5]. In the present study, 44.5% of non-survivors had at least one of comorbidities while the prevalence of any comorbidities was 23.2% in survivors, raising awareness of the need for earlier monitoring and greater supportive care in this vulnerable group. In accordance with previous observations, we showed that the prevalence of DM and CVD in non-survivors was higher than in survivors. In a retrospective, multicentre cohort study in Wuhan, China, which has been conducted on 191 patients with laboratory-confirmed COVID-19, the risk factors associated with in-hospital death had been explored [15]. The prevalence of DM and CVD was recorded respectively 19% and 8% in hospitalized patients and 31% and 24% in non-survivors [15], indicating a higher prevalence of comorbidities in infected non-survivors in agreement with our findings. Moreover, DM has been declared as one of the most common comorbidities in deceased COVID-19 patients in some of the European countries [18].

The present study is among the first studies with the approach of exploring the risk factors of mortality in COVID-19 patients. We included different diagnostic approaches for COVID-19 diagnosis, firstly based on the clinical symptoms and chest CT scan as well as laboratory confirmation tests. Furthermore, we performed subgroup analyses for gender and age groups. Besides, our study has some limitations. Firstly, due to the retrospective design of the study, some laboratory tests were not collected for all patients, and these missing data might lead to bias of clinical characteristics. Considering that cases with more severe disease hospitalized, the percentage of mortality in our study cannot reflect the true fatality ratio of COVID-19 and might limit the interpretation of our findings. Moreover, the existence of DM and CVD was self-reported data, which should be cautiously interpreted because of probable reporting bias. It should be noted that we had no access to details about diabetic patients such as duration of diabetes and kind of treatments so these issues were not considered in our analysis, although we follow the same protocol for controlling DM all over the province. We also missed the effects of some comorbidities like obesity on the mortality rate of COVID-19. Moreover, HbA1c was not measured in all diabetic patients in our study, and therefore the glycemic control level was unclear in our patients.

## Conclusions

Our findings support the hypothesis that diabetic patients have an increased risk of in-hospital mortality following COVID-19. Our findings also suggest lymphocyte count, creatinine and CRP concentrations might be significant predictors for the death of COVID-19 in these patients.

## Supplementary information

**Additional file 1.** Additional tables and figure.

## Data Availability

The datasets used and analysed during the current study are available from the corresponding author on reasonable request.

## Abbreviations

ACE2: Angiotensin-converting enzyme 2
ALT: Alanine transaminases
AST: Aspartate transaminases
Is: Confidence intervals
COVID-19: Coronavirus disease 2019
CRP: C-reactive protein
CT: Chest computed tomography
CVD: Cardiovascular diseases
DM: Diabetes mellitus
Esr: Erythrocyte sedimentation rate
Hb: Hemoglobin
IQR: Inter quartile range
MERS: Middle East respiratory syndrome
LDH: Lactate dehydrogenase
OR: Odds ratios
PT: Prothrombin time
RT-PCR: Real-time polymerase chain reaction
SARS-CoV-2: Severe acute respiratory syndrome coronavirus 2
WBC: White blood cell
