# Supplementary material for: Risk and predictors of in-hospital mortality from COVID-19 in patients with diabetes and cardiovascular disease
Source: Diabetol Metab Syndr. 2020 Jul 6;12:57. doi: 10.1186/s13098-020-00565-9 (PMC7338100; doi:10.1186/s13098-020-00565-9)
Supplement: Supplementary file 1 [file 13098_2020_565_MOESM1_ESM.doc]

Additional file 1: Table S1: Frequency of the comorbidities in laboratory-confirmed patients with COVID-19, overall and by survival status  in Laboratory Confirmed patients		Characteristics	Total
% (N)	Non-survivors
% (N)	Survivors
% (N)	P-value		DM (all)	9.2%  (130)	16.6% (28)	8.2% (102)	<0.001		Only DM	5.4% (76)	9.5% (16)	4.8% (60)	0.012								CVD (all)	9.5% (134)	16.6% (28)	8.5% (106)	0.001		Only CVD	5.3% (75)	9.5% (16)	4.7% (59)	0.010								DM or CVD	16.1% (227)	28.4% (48)	14.4% (179)	<0.001		Any comorbidity 	23.9%(338)	46.2%(78)	20.9%(260)	<0.001		CVD= Cardiovascular diseases. DM= Diabetes mellitus.
DM (all): DM with or without other comorbidities. "Only DM": DM without other comorbidities. CVD (all):  CVD with or without other comorbidities. "Only CVD": CVD without other comorbidities. "Any comorbidity": The presence of any comorbidity		 
.

Additional file 1: Table S2: Risk of  mortality due to comorbidities in Laboratory Confirmed patients  with COVID 19: Logistic regression model			OR (95% CI)		Characteristics	Model Ia	Model IIb	Model IIIc		DM (all)	2.22 (1.41-3.50) *	2.21 (1.40-3.48) *	1.76 (1.09 -2.84) *		Only DM	2.06 (1.16-3.67) *	2.05 (1.15-3.64) *	1.97 (1.07-3.63) *							CVD	2.13 (1.36-3.35) *	2.12(1.35 –3.33) *	1.05 (0.64-1.71)		Only CVD	2.10 (1.18-3.74) *	2.09(1.17 -3.73) *	0.93 (0.50-1.73)							DM or CVD	2.36 (1.63-3.41) *	2.35 (1.62 -3.40) *	1.40 (0.94-2.09)		Any comorbidity	3.24 (2.32-4.52) *	3.24(2.32 –4.52) *	2.14 (1.50-3.04) *		a:crude, b: adjusted for gender, c: additionally adjusted for age and  laboratory  tests; * P < 0.05, 
CVD= Cardiovascular diseases. DM= Diabetes mellitus.
DM (all): DM with or without other comorbidities. "Only DM": DM without other comorbidities. CVD (all):  CVD with or without other comorbidities. "Only CVD": CVD without other comorbidities. "Any comorbidity": The presence of any comorbidity.		







           

	A	B
Additional file 1: Figure S1: Forest plot of the multivariate association between  the comorbidities and in-hospital mortality by  age group  (A; adjusted for age and gender) and gender (B; adjusted for age in year), in laboratory-confirmed patients with COVID-19 infection
DM (all): DM with or without other comorbidities. "Only DM": DM without other comorbidities. CVD (all):  CVD with or without other comorbidities. "Only CVD": CVD without other comorbidities. "Any comorbidity": The presence of any comorbidity.
